# Immunotherapy for Prostate Cancer: Where We Are Headed

**DOI:** 10.3390/ijms18122627

**Published:** 2017-12-05

**Authors:** Giuseppe Schepisi, Alberto Farolfi, Vincenza Conteduca, Filippo Martignano, Delia De Lisi, Giorgia Ravaglia, Lorena Rossi, Cecilia Menna, Salvatore Roberto Bellia, Domenico Barone, Roberta Gunelli, Ugo De Giorgi

**Affiliations:** 1 Department of Medical Oncology, Istituto Scientifico Romagnolo per lo Studio e la Cura dei Tumori (IRST) IRCCS, Via P. Maroncelli 40, 47014 Meldola, Italy; alberto.farolfi@irst.emr.it (A.F.); vincenza.conteduca@irst.emr.it (V.C.); lorena.rossi@irst.emr.it (L.R.); cecilia.menna@irst.emr.it (C.M.); ugo.degiorgi@irst.emr.it (U.D.); 2 Biosciences Laboratory, Istituto Scientifico Romagnolo per lo Studio e la Cura dei Tumori (IRST) IRCCS, Via P. Maroncelli 40, 47014 Meldola, Italy; filippo.martignano@gmail.com; 3 Medical Oncology Department, Campus Bio-Medico University, Via Alvaro del Portillo 200, 00128 Rome, Italy; d.delisi@unicampus.it; 4 Unit of Biostatistics and Clinical Trials, Istituto Scientifico Romagnolo per lo Studio e la Cura dei Tumori (IRST) IRCCS, Via P. Maroncelli 40, 47014 Meldola, Italy; giorgia.ravaglia@irst.emr.it; 5 Radiotherapy Unit, Istituto Scientifico Romagnolo per lo Studio e la Cura dei Tumori (IRST) IRCCS, Via P. Maroncelli 40, 47014 Meldola, Italy; salvatore.bellia@irst.emr.it; 6 Radiology Unit, Istituto Scientifico Romagnolo per lo Studio e la Cura dei Tumori (IRST) IRCCS, Via P. Maroncelli 40, 47014 Meldola, Italy; domenico.barone@irst.emr.it; 7 Urology Unit, Forlì Hospital, Romagna Local Health Service, 47100 Forlì, Italy; r.gunelli@ausl.fo.it

**Keywords:** prostate cancer, immunotherapy, vaccines, CTLA4, PD1, PD-L1, antibodies

## Abstract

Prostate cancer is one of the most common malignant neoplasms in men worldwide, and is the fifth cause of cancer-related death. In recent years, a new generation of therapies have been approved for the management of metastatic disease. Moreover, the development of new immunotherapeutic drugs has become a novel frontier for the treatment of several tumor types; to date, numerous studies have investigated their potential activity, including in prostate cancer. In this article, we discuss the role of emerging immunotherapeutic drugs in prostate cancer patients.

## 1. Introduction

In recent years, advances in clinical research have led to the approval of several treatments for stage IV prostate cancer (PCa): two next-generation androgen receptor (AR)-directed therapies (abiraterone acetate and enzalutamide), a chemotherapeutic agent (cabazitaxel) and a radiopharmaceutical agent (radium-223). To date, Sipuleucel-T is the only immunotherapeutic intervention approved by the Food and Drug Administration (FDA) to treat advanced PCa; however, many other vaccines are currently under investigation. In this article, we discuss the role of emerging immunotherapeutic drugs in PCa patients.

A comprehensive literature search was conducted, including PubMed, Medline and Embase platforms, to identify manuscripts reporting on programmed death 1 (PD-1) and programmed death ligand 1 (PD-L1) expression and checkpoint inhibition in PCa.

## 2. Rationale for Immunotherapies in PCa

PCa is generally considered a slow-growing tumor, which may allow adequate time for an immunotherapy agent to activate the immune system. Both the innate and the adaptive part of the immune system are thought to be involved in host defense mechanisms against PCa. In the last few years, several studies have investigated the role played, if any, by the immune system in controlling/eradicating PCa. Flammiger et al. [1] found a correlation between intratumoral T, but not B, lymphocytes and PCa. In particular, a higher percentage of regulatory T-cells (Tregs) was more predominant in higher stages of PCa. Moreover, tumor-infiltrating cytotoxic lymphocytes express higher levels of Programmed cell death protein 1 (PD1, an indicator of T-cell exhaustion) [2,3].

Other studies have found that B lymphocytes promote PCa progression through the activation of IKKα, STAT3 and BMI1 in castration-resistant PCa cells [4,5]. The presence of CD20^+^ lymphocytes has been described in PCa, with higher concentrations in malignant than benign tissue [6].

The role of Neutrophil-to-lymphocyte ratio (NLR) in PCa is still uncertain. Some studies have described a correlation between higher NLR and poor prognosis in post-docetaxel PCa patients [7]. In other studies, NLR was an effective predictor of Prostate Specific Antigen (PSA), but not of clinical response after chemotherapy [8]. Fujita el al., demonstrated that a higher NLR is an effective predictor of a benign prostate biopsy [9].

Some studies have also described the role of tumor-infiltrating Macrophage (TAM) count in PCa. One study confirmed its prognostic role in clinical outcome, but other studies have concentrated this correlation to specific TAM subclasses (e.g., M2-type macrophages [10]) and to specific categories of patients (only in the control, instead of in the androgen-deprivation treated (ADT) group [11]).

Furthermore, PCa has many well-described tumor-associated antigens (TAAs), which may be ideal targets for immunotherapy. Examples of TAA for PCa include PSA, prostatic acid phosphatase (PAP), and prostate-specific membrane antigen (PSMA) [12].

Alteration on DNA repair genes represents a significant genomic defect associated with high risk of developing gynecological malignancies [13]. Recently, a whole exome and transcriptome analysis, performed on 150 biopsies from primary or metastatic PCa (mPCa) tissue, showed that germline or somatic aberration in DNA repair genes, such as *AR*, *ETS* genes, *TP53*, *PTEN*, *PIK3CA/B*, R-spondin, *BRAF/RAF1*, *APC*, β-catenin, *ZBTB16/PLZF*, *BRCA2*, *BRCA1*, and *ATM*, are representative in both primary and advanced PCa [14,15].

Although the prevalence of germline mutations in DNA-repair genes among men with localized PCa is quite low, the incidence among men with mPCa seems significantly higher (odds ratio, 5.3; 95% CI, 1.9 to 20.2; *p* < 0.001) [16]. It is not yet clear if this difference is correlated with an acquired mutational load due to therapy exposure, or with an intrinsic PCa primary aggressiveness. However, it was demonstrated that PCa with germline Breast Related Cancer Antigen (*BRCA*)*1/2* mutations were more frequently associated with Gleason ≥ 8 (*p* = 0.00003), T3/T4 stage (*p* = 0.003), nodal involvement (*p* = 0.00005), and metastases at diagnosis (*p* = 0.005) than in non-carriers PCa, resulting in reduced cancer-specific survival [17].

The alteration in DNA repair pathways has been of interest to the scientific community since demonstrating that a PD-1 inhibitor was active against colorectal cancer with deficiency in mechanisms of mismatch repair. In fact, in this tumor subtype, a rich lymphocyte tumor infiltrate has been detected, suggesting a rationale for inhibiting PD-1 and PDL1 pathway [18]. Even defects in *BRCA* mutation carriers may be a target for immune-checkpoint inhibitors, since tumors harboring *BRCA* mutations, or with a *BRCA*-like phenotype, may be associated with a higher probability of producing new neoantigens and/or to possessing a higher mutational load, thus stimulating an immune response, as suggested by the observations of a more pronounced lymphocyte infiltration in ovarian cancer [19,20]. However, although in ovarian cancer the presence of a mutation in *BRCA1/2* is associated with a better prognosis [21], germline *BRCA2* mutations seems to be an independent factor for poor prognosis in PCa, but the reason of this poor outcome is still unclear [22].

## 3. Cancer Vaccines

The rationale behind vaccines in tumors is to induce a strong and effective immune response against tumor-related antigens, which can eradicate tumors. Several approaches to vaccine-based immunotherapy have been studied, including autologous or heterologous cell or peptide vaccines, viral- and DNA-based vaccines.

Sipuleucel-T is a vaccine derived from the co-culture of the patient’s own PBMC with a fused granulocyte macrophage colony-stimulating factor and prostatic acid phosphatase (GM-CSF-PAP) protein [23,24]. The aim of this process is to activate the APCs, and to start the immune response. Sipuleucel has been used in PCa patients in three trials.

In the first two trials, (D9901 and D9902A), a total of 225 “hormone-refractory” PCa patients received Sipuleucel-T infusions or placebo every 2 weeks. Median time to progression (TTP) was not reached, but a statistically significant Overall Survival (OS) benefit of 4.3 months was shown, warranting further studies on Sipuleucel-T in PCa patients. The IMPACT trial, a phase III study, recruited 512 PCa patients to receive Sipuleucel-T or placebo a 22% reduction in the risk of death was shown, with a benefit of 4.1-month in OS. Adverse events with new immunotherapeutic treatment were often mild and manageable [25].

In a retrospective analysis Schellhammer et al. [26] showed that patients with lower baseline PSA level present an improvement in OS of 13 months compared to patients with higher PSA baseline level, where only a 2.8 month benefit was observed. This analysis demonstrated that Sipuleucel T has a better efficacy in patients with lower tumor burden. However, it has been speculated that removal of a large part of circulating lymphocytes by leukapheresis could negatively impact patients’ immune systems [27].

The results obtained in this trial have encouraged additional trials, which are currently ongoing, using Sipuleucel T vaccine approach in combination with other approved drugs, such as abiraterone acetate, enzalutamide, radium-223 (NCT01487863, NCT01981122, NCT02463799, NCT01832870); another trial is evaluating Sipuleucel T in combination with CTLA4-inhibitor Ipilimumab (NCT01804465).

PROSTVAC-VF is a poxvirus-based vaccine consisting of a recombinant vaccinia vector followed by multiple booster vaccinations that induces PSA immune responses through genetically modified vaccinia and fowlpox encoding PSA and 3 costimulatory proteins, B7.1, ICAM-1 and LFA-3 (designated TRICOM™). In a phase I study, 19/33 patients treated with PROSTVAC-VF achieved a PSA reduction during the study, and 9/33 patients had a PSA stabilization for 11–21 months after vaccination [28]. In another phase I trial, 4/10 patients treated with PROSTVAC-VF had a PSA stabilization during the 8-week study period [29]. In a Phase II study, 125 patients treated with PROSTVAC achieved a higher 3-year-OS than the control group (30% vs. 17%) [30]. A phase III study (BNIT-PRV-301-PROSPECT trial) was completed in asymptomatic or minimally symptomatic mPCa patients. 1298 patients were enrolled and randomized into 3 arms (PROSTVAC-V/F-TRICOM + GM-CSF; PROSTVAC-V/F-TRICOM + GM-CSF placebo; placebo alone); the results of this trial are awaited (NCT01322490).

ProstAtak^®^ (AdV-tk) is a new vaccine approach, known as Gene-Mediated Cytotoxic Immunotherapy (GMCI). The effect of the vaccine is mediated by intra-tumoral delivery of a Herpes virus thymidine-kinase gene (AdV-tk), inserted in an adenoviral vector, followed by systemic anti-herpetic prodrug (valacyclovir). This vaccine is combined with standard surgery and radiation [31]. Based on the interesting results obtained in a previous phase I-II trial, [32] a phase III trial has started, which is still ongoing, to evaluate the effectiveness of ProstAtak^®^ immunotherapy in combination with radiation therapy for intermediate- to high-risk localized PCa patients. (NCT01436968).

PAN-301-1 is a Human aspartyl-asparaginyl-β-hydroxylase (HAAH)-directed nanoparticle vaccine. HAAH, also known as aspartate-β-hydroxylase, is a transmembrane protein that catalyzes the hydroxylation of aspartyl and asparaginyl residues in epidermal growth factor-like domains of Notch and homologs. It has been described in several tumor types, including PCa. In this vaccine, HAAH peptides are fused at the head protein gpD of phage lambda, which carries 200–300 copies of the gpD protein. A phase I trial to evaluate safety and immunogenicity of the PAN-301-1 vaccine biochemically-relapsed PCa patients (NCT03120832) is ongoing.

## 4. Immune-Checkpoint Inhibitors

### 4.1. Anti-CTLA4 Antibodies

#### 4.1.1. Ipilimumab

**Figure 1:**
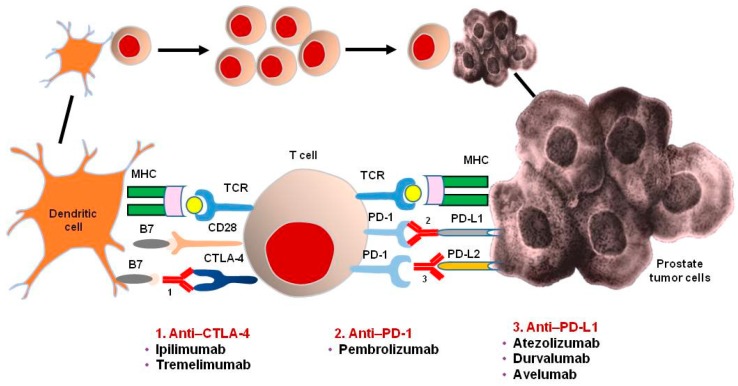
Immune Checkpoint Inhibitors based on CTLA-4 and PD-1/PD-L1 blockade in prostate cancer. Abbreviations. MHC, major histocompatibility complex; TCR, T-cell receptor. Black continuous line: indicates an enlargement of the figure to which it refers; Black arrow line indicates an activation of the previous cell(s).

Ipilimumab is a fully human monoclonal immunoglobulin G1 antibody that binds cytotoxic T-lymphocyte antigen 4 (CTLA4) (Figure 1). This drug provided significant survival benefit in two phase III studies of advanced melanoma, as a single agent and in combination with dacarbazine, with 20% of patients experiencing long-term survival [33,34]. Ipilimumab has also been evaluated in PCa patients (Table 1).

In 2013, a multicenter phase I/II trial explored the efficacy and safety of Ipilimumab in combination or alone with radiotherapy in 71 PCa patients. A clinical benefit (in terms of complete response and stable disease) and a PSA decline >50% was observed in seven and eight patients, respectively [35]. In 2014, a phase III study (CA184-043) tested single-agent ipilimumab 10 mg/kg after bone-directed radiotherapy (8 Gy in one fraction) in mPCa patients pretreated with docetaxel. Ipilimumab demonstrated antitumor activity, with an improvement in progression-free survival (PFS) and PSA responses; but no improvement in OS was found [36]. Exploratory analysis of this trial suggested a role of Ipilimumab in increasing OS only in a cohort of PCa patients with favorable prognostic features.

More recently, a randomized phase III study (CA184-095) investigated ipilimumab versus placebo in PCa patients without visceral metastases. Median PFS in patients treated with Ipilimumab (5.6 months) were longer than the placebo cohort (3.8 months), and the same results were found for the PSA response rate. However, no OS improvement was observed between the two arms [37]. Moreover, this study did not show significant differences in terms of efficacy between patient subgroups (with or without visceral metastases). Despite the fact that remissions were rare in both phase III trials, some cases of long-term complete responses with this drug have been described in the literature [38]. Unfortunately, to date, the lack of predictive biomarkers limits the selection of patients who would benefit from treatment with checkpoint inhibitors.

Several studies are currently ongoing to evaluate the efficacy of Ipilimumab in combination with hormonal therapy. The rationale of this combination resides in the ability of hormonal therapy to modulate immune system activity [39].

In a phase II study, the combination of Ipilimumab 3 mg/kg and androgen ablation demonstrated a more frequent incidence of undetectable PSA at 3 months than androgen ablation alone (55% vs 38%). Two additional studies are currently ongoing to evaluate the same combination treatment: the first trial (NCT01498978) is a phase II study evaluating Ipilimumab with ADT in patients with incomplete response to hormonal therapy alone. The second trial is a phase I/II study of combination between Ipilimumab and Abiraterone acetate in chemo- and immunotherapy-naïve mPCa patients (NCT01688492).

#### 4.1.2. Tremelimumab

Tremelimumab (formerly ticilimumab, CP-675,206) is a fully human IgG2 monoclonal antibody. It acts by blocking the binding of the ligands B7.1 and B7.2 to CTLA-4, resulting in inhibition of B7-CTLA-4-mediated downregulation of T-cell activation.

To date, some studies have evaluated its efficacy against PCa, in neoadjuvant and in recurrent settings. A phase I dose-escalation trial is evaluating the role of different doses of Tremelimumab in combination with androgen deprivation in PCa patients with D0 disease; a roll-over study is also currently ongoing for patients with PCa (NCT00378482). 

### 4.2. Anti-PD1 Antibodies

#### Pembrolizumab

Pembrolizumab (MK-3475) is a potent and highly selective humanized monoclonal antibody (mAb) of the IgG4/kappa isotype designed to directly block the interaction between the programmed cell death-1 receptor (PD-1) and its ligands, PD-L1 and PD-L2.

Ongoing Clinical Trials (Table 2) In KEYNOTE-028 trial, a non-randomized, phase 1b trial, patients with PD-L1 positive mPCa have been treated with pembrolizumab monotherapy. Of the 23 patients enrolled in the PCa cohort, all had prior treatment with docetaxel and targeted endocrine therapy. Three confirmed partial responses were observed, with an overall response rate (ORR) of 13%; median duration of response was 59 weeks, and stable disease rate was 39%. The responses were durable, and treatment was well tolerated [40]. 

To further evaluate the signal of activity observed in this study, the KEYNOTE-199 trial was designed; this is a nonrandomized, open-label, multinational phase II trial of pembrolizumab in previously treated metastatic PCa patients. A core or excisional biopsy of a tumor lesion and/or tissue from an archival tissue sample to evaluate for PD-L1 expression by IHC is needed for participation in this trial. Patients with PD-L1 positive and PD-L1 negative tumors will be enrolled in the trial. The prediction of response to anti-PD-1 therapy is based on the results from Topalian et al. In their study, an ORR was shown in 9/25 (36%) of PD-L1-expressing tumors, versus 0/17 responses shown in PD-L1-negative tumors [41]. KEYNOTE-199 is currently ongoing.

### 4.3. Anti-PDL1 Antibodies

#### 4.3.1. Atezolizumab

Atezolizumab (MPDL3280) is a fully humanized, engineered, IgG1 antibody that binds PDL1. It has shown promising results in the treatment of a number of different cancers, including melanoma, lung, bladder and renal cancer. In 2016, FDA approved atezolizumab as second-line for patients with locally advanced or metastatic urothelial cancer after failure of a first-line chemotherapy.

At present, four studies are evaluating this drug in mPCa. In phase Ib, open label study, Atezolizumab in combination with radium-223 dichloride is evaluated in PCa patients after treatment with an androgen pathway inhibitor (NCT02814669).

Another phase Ib trial compares the safety and tolerability of sequential atezolizumab followed by sipuleucel-T (Arm 1) versus sipuleucel-T followed by atezolizumab (Arm 2) in patients who have asymptomatic or minimally symptomatic chemo-naïve patients with mPCa (NCT03024216). 

An open-label, multicohort, phase II trial is evaluating Atezolizumab in patients with advanced solid tumors, including PCa (NCT02458638). 

IMbassador250, a phase III randomized trial is evaluating the combination of atezolizumab with enzalutamide compared with enzalutamide alone in PCa patients after failure of the ADT and failure of a taxane regimen (NCT03016312). 

#### 4.3.2. Durvalumab

Durvalumab (MEDI4736) is a human IgG1 anti-PD-L1 antibody, which was granted breakthrough therapy designation by the FDA in February 2016 for urothelial bladder cancer patients whose tumor has progressed during or after a standard platinum-based regimen. It is also currently under investigation for the treatment of several tumor types, including mPCa.

At present, four studies are evaluating Durvalumab in mPCa. A phase II trial compares Durvalumab alone versus combination treatment with anti-CTLA4 Tremelimumab in patients with mPCa (NCT02788773).

The same combination is being evaluated in a phase I/II trial in association with the tumor microenvironment (TME) modulator polyICLC, a TLR3 agonist, in subjects with advanced, measurable, biopsy-accessible cancers, including mPCa (NCT02643303).

A phase I/II study is ongoing to evaluate safety and efficacy of Durvalumab in combination with Olaparib and/or Cediranib for advanced solid tumors (including PCa) (NCT02484404). Another phase II trial studies Durvalumab in mPCa patients. A set of tests are planned, including mismatch repair gene mutational status plus mutational load and PDL1 expression assessed by IHC and transcript profiling (NCT02966587).

#### 4.3.3. Avelumab

Avelumab (MSB0010718C) is a fully human IgG1 antibody against PDL1. To date, PCa patients have been treated with Avelumab only in the context of a phase I study (NCT01772004).

## 5. Perspectives

PCa is a heterogeneous disease with aggressive variants with specific features including neuroendocrine differentiation [42,43,44] and germline *BRCA1/2* mutations. The role of potential biomarkers involved in immunological activation mechanisms in different PCa subtypes is under investigation.

According to Schumacher and Schreiber [45], PCa is a tumor with a relatively low mutational burden, similar to pancreatic cancer. Perhaps more data could be obtained from the analysis of specific cancer subtypes, in particular from aggressive variant PCa (AVPC). These variants are a biologically distinct subset that shares molecular and therapeutic phenotype of the small cell PCa, characterized by combined defects in various genes, including *TP53*, *RB1*, *PTEN* [46]. At present, a phase II study is evaluating the role of anti-PD1 Nivolumab in PCa patients with defects in DNA repair mechanisms (NCT03040791).

Unfortunately, a specific biomarker to guide therapy choices is not available, to date. The absence of a specific biomarker could also potentially explain the lack of efficacy observed in several studies. The role of PD-L1 expression as a predictive biomarker of response in case of treatment with PD-1/PDL1 antibodies has been shown in lung cancer patients, and is under investigation in several tumors [47,48]. Not only PD-L1 expression, but also other parameters, e.g., NLR [49,50] or Immune-Inflammation Index [51], are under investigation in several tumor types, including also PCa.

In PCa, studies evaluating the role of soluble markers in blood samples are currently ongoing [52].

Finding more specific biomarkers could be useful not only for selecting more appropriate therapy for every single patient, but also to respond to several unanswered questions: timing of immunotherapies, possibility of withdrawing therapy and rechallenge after progression.

## 6. Conclusions

Since the approval of Sipuleucel-T in 2010, Immunotherapy has become an intriguing option also for PCa patients; conversely, PCa represents a very interesting histology for the development of immunotherapeutic agents, due to its intrinsic immune-stimulating characteristics; unfortunately, in trials conducted with anti-CTLA4, in particular Ipilimumab, a significant impact on outcomes has not been observed. At present, attention is mainly focused on anti-PD1/PDL1 inhibitors, with several studies evaluating the role of these molecules against PCa currently ongoing. It will be useful to define the precise setting of treatment, considering immune therapy not only as a monotherapy, but also as a part of combination with other therapies (chemotherapy, hormonal agents, radiotherapy, other check-point inhibitors). The other aim for future studies is to find a specific biomarker that can select potentially responsive patients to immunotherapeutic agents.

## Figures and Tables

**Table 1:** Phase 2 or 3 studies evaluating anti-CTLA4 antibodies in PCa.

Agent	Phase	Population	Study Arms	Enrollment/Expected Enrollment	Recruitment Status	Primary Outcome	NCT Number
Ipilimumab after bone-directed radiotherapy	3	mCRPC patients pretreated with docetaxel	Ipilimumab vs. placebo	988	Completed	OS	NCT00861614
Ipilimumab	3	chemonaïve mCRPC patients	Ipilimumab vs. placebo	837	Completed	OS	NCT01057810
Ipilimumab + ADT	2	mCRPC patients	Ipilimumab + ADT	10	Active, not recruiting	undetectable PSA ( ≤0.2 ng/mL) up to 5 years	NCT01498978
Ipilimumab + Leuprolide Acetate	2	Neoadjuvant setting	Ipilimumab + Leuprolide Acetate	19	Completed	immunological variables (T cell ratio, NY-ESO-1 antibodies, ALC, CD4^+^ ICOS^+^ and CD8^+^ ICOS^+^ T cells)	NCT01194271
Tremelimumab	2	Rollover study for PCa (or other cancers) patients previously treated with Tremelimumab	Tremelimumab	38	Active, not recruiting	Safety, Tumor status: AWD or NED, OS	NCT00378482

OS = overall survival; NR = Not reported; ADT = androgen deprivation therapy; ALC = absolute lymphocyte count; AWD = alive with disease; NED = no evidence of disease.

**Table 2:** Phase 1 to 3 studies evaluating anti-PD1/PDL1 antibodies in PCa.

Drugs	Phase	Population	Study Arms	Enrollment/Expected Enrollment	Recruitment Status	Primary Outcome	NCT Number
Nivolumab	2	PCa pretreated with DNA repair defects	Nivolumab	29	Active, not recruiting	PSA response rate	NCT03040791
Pembrolizumab	1b	PD-L1 positive mCRPC patients	Pembrolizumab	477	Active, not recruiting	OR	NCT02054806
Pembrolizumab	2	mCRPC patients pretreated with chemotherapy	Pembrolizumab	250	Recruiting	OR	NCT02787005
Atezolizumab + radium-233 dichloride	1b	mCRPC patients	Atezolizumab + radium-233 dichloride (Concurrent vs. Staggered 28-Day Run-in vs. Staggered 56-day run-in)	45	Recruiting	DLTs, AEs, OR	NCT02814669
Atezolizumab + sipuleucel-T	1b	asymptomatic or minimally symptomatic chemo-naïve mCRPC patients	Atezolizumab before and after sipuleucel-T	34	Recruiting	AEs, changes in vital signs and clinical laboratory results	NCT03024216
Atezolizumab	2	Patients with advanced solid tumors (including PCa)	Atezolizumab	725	Recruiting	NPR	NCT02458638
Atezolizumab + enzalutamide	3	mCRPC patients progressed on androgen-synthesis inhibitor, untreatable with taxanes	Atezolizumab + enzalutamide vs. enzalutamide	558	Recruiting	OS	NCT03016312
Durvalumab + Tremelimumab	2	mCRPC patients	Durvalumab alone vs. Durvalumab + Tremelimumab	74	Recruiting	OR	NCT02788773
Durvalumab + Tremelimumab + polyICLC	1/2	advanced, measurable, biopsy-accessible cancers (including mCRPC)	IV Durvalumab + IT/IM polyICLC vs. IV Durvalumab + IV Tremelimumab + IT/IM polyICLC vs. IV Durvalumab + IT Tremelimumab + IT/IM polyICLC	102	Recruiting	Recommended dose, OR, PFS, OS.	NCT02643303
Durvalumab + Olaparib, Durvalumab + Cediranib	1	Advanced solid tumors (including PCa)	Durvalumab + Olaparib vs. Durvalumab + Cediranib vs. Durvalumab + Olaparib + Cediranib	338	Recruiting	Recommended dose, safety	NCT02484404
Durvalumab	2	mCRPC patients	Durvalumab	28	Not yet recruiting	OR	NCT02966587
Avelumab	1	Advanced solid tumors (including mCRPC)	Avelumab	1706	Recruiting	DLTs, OR	NCT01772004

OR = overall response; DLTs = dose-limiting toxicities; AEs = adverse events; NPR = non-progression rate; OS = Overall Survival; PFS = Progression Free Survival; IV = intra venous administration; IT = intra tumoral administration.
